# Normal Laws for Two Entropy Estimators on Infinite Alphabets

**DOI:** 10.3390/e20050371

**Published:** 2018-05-17

**Authors:** Chen Chen, Michael Grabchak, Ann Stewart, Jialin Zhang, Zhiyi Zhang

**Affiliations:** Department of Mathematics and Statistics, University of North Carolina at Charlotte, Charlotte, NC 28223, USA

**Keywords:** entropy, nonparametric estimator, Miller–Madow estimator, jackknife estimator, asymptotic normality, Primary 62F10, 62F12, 62G05, 62G20

## Abstract

This paper offers sufficient conditions for the Miller–Madow estimator and the jackknife estimator of entropy to have respective asymptotic normalities on countably infinite alphabets.

## 1. Introduction

Let $X=\{\ell_{k};k\geq 1\}$ be a finite or countably infinite alphabet, let $p=\{p_{k};k\geq 1\}$ be a probability distribution on X, and define $K=\sum_{k\geq 1}1\left[p_{k}>0\right]$, where 1[·] is the indicator function, to be the effective cardinality of X under p. An important quantity associated with p is entropy, which is defined by [1] as

(1) $$H=-\sum_{k\geq 1}p_{k}\ln p_{k}.$$

Here and throughout, we adopt the convention that 0ln0=0.

Many properties of entropy and related quantities are discussed in [2]. The problem of statistical estimation of entropy has a long history (see the survey paper [3] or the recent book [4]). It is well-known that no unbiased estimators of entropy exist, and, for this reason, much energy has been focused on deriving estimators with relatively little bias (see [5] and the references therein for a discussion of some (but far from all) of these). Perhaps the most commonly used estimator is the plug-in. Its theoretical properties have been studied going back, at least, to [6], where conditions for consistency and asymptotic normality, in the case of finite alphabets, were derived. It would be almost fifty years before corresponding conditions for the countabe case would appear in the literature. Specifically, consistency, both in terms of almost sure and $L^{2}$ convergence, was verified in [7]. Later, sufficient conditions for asymptotic normality were derived in two steps in [3,8].

Despite a simple form and nice theoretical properties, the plug-in suffers from large finite sample bias, which has led to the development of modifications that aim to reduce this bias. Two of the most popular are the Miller–Madow estimator of [6] and the jackknife estimator of [9]. Theoretical properties of these have not been studied, as extensively, in the literature. In this paper, we give sufficient conditions for the asymptotic normality of these two estimators. This is important for deriving confidence intervals and hypothesis tests, and it immediately implies consistency (see e.g., [4]).

We begin by introducing some notation. We say that a distribution $p=\{p_{k};k\geq 1\}$ is uniform if and only if its effective cardinality K<∞ and for each k=1,2,⋯ either $p_{k}=1/K$ or $p_{k}=0$. We write f∼g to denote $\lim_{n\to \infty }f\left(n\right)/g\left(n\right)=1$ and we write f=O(g(n)) to denote ${lim sup}_{n\to \infty }\left|f(n)/g(n)\right|<\infty$. Furthermore, we write $\overset{L}{\to }$ to denote convergence in law and $\overset{p}{\to }$ to denote convergence in probability. If *a* and *b* are real number, we write a∨b to denote the maximum of *a* and *b*. When it is not specified, all limits are assumed to be taken as n→∞.

Let $X_{1},\cdots ,X_{n}$ be independent and identically distributed (iid) random variables on X under p. Let $\left\{Y_{k};k\geq 1\right\}$ be the observed letter counts in the sample, i.e., $Y_{k}=\sum_{i=1}^{n}1\left[X_{i}=\ell_{k}\right]$, and let $\hat{p}=\left\{{\hat{p}}_{k};k\geq 1\right\}$, where ${\hat{p}}_{k}=Y_{k}/n$, be the corresponding relative frequencies. Perhaps the most intuitive estimator of *H* is the plug-in, which is given by

(2) $$\hat{H}=-\sum_{k\geq 1}{\hat{p}}_{k}\ln{\hat{p}}_{k}.$$

When the effective cardinality, *K*, is finite, [10] showed that the bias of $\hat{H}$ is

(3) $$E\left(\hat{H}\right)-H=-\frac{K-1}{2n}+\frac{1}{12n^{2}}\left(1-\sum_{k=1}^{K}\frac{1}{p_{k}}\right)+O\left(n^{-3}\right).$$

One of the simplest and earliest approaches aiming to reduce the bias of $\hat{H}$ is to estimate the first order term. Specifically, let $\hat{m}=\sum_{k\geq 1}1\left[Y_{k}>0\right]$ be the number of letters observed in the sample and consider an estimator of the form,

(4) $${\hat{H}}_{M\;M}=\hat{H}+\frac{\hat{m}-1}{2n}.$$

This estimator is often attributed to [6] and is known as the Miller–Madow estimator. Note that, for finite *K*,

$$E\left(\frac{\hat{m}-1}{2n}\right)=\frac{K-1}{2n}-\frac{\sum_{k}{\left(1-p_{k}\right)}^{n}}{2n}.$$

Since $\sum_{k}{\left(1-p_{k}\right)}^{n}\leq K{\left(1-p_{\wedge }\right)}^{n}$, where $p_{\wedge }=\min\left\{p_{k}:p_{k}>0\right\}$, decays exponentially fast, it follows that, for finite *K*, the bias of ${\hat{H}}_{M\;M}$ is

$$E\left({\hat{H}}_{M\;M}\right)-H=\frac{1}{12n^{2}}\left(1-\sum_{k=1}^{K}\frac{1}{p_{k}}\right)+O\left(n^{-3}\right).$$

Among the many estimators in the literature aimed at reducing bias in entropy estimation, the Miller–Madow estimator is one of the most commonly used. Its popularity is due to its simplicity, its intuitive appeal, and, more importantly, its good performance across a wide range of different distributions including those on countably infinite alphabets. See, for instance, the simulation study in [5].

The jackknife entropy estimator is another commonly used estimator designed to reduce the bias of the plug-in. It is calculated in three steps:

1. for each i∈{1,2,⋯,n} construct ${\hat{H}}^{\left(i\right)}$, which is a plug-in estimator based on a sub-sample of size n-1 obtained by leaving the *i*th observation out;
2. obtain ${\hat{H}}_{\left(i\right)}=n\hat{H}-\left(n-1\right){\hat{H}}^{\left(i\right)}$ for i=1,⋯,n; and then
3. compute the jackknife estimator
  (5) $${\hat{H}}_{J\;K}=\frac{\sum_{i=1}^{n}{\hat{H}}_{\left(i\right)}}{n}.$$

Equivalently, (5) can be written as

(6) $${\hat{H}}_{J\;K}=n\hat{H}-\left(n-1\right)\frac{\sum_{i=1}^{n}{\hat{H}}^{\left(i\right)}}{n}.$$

The jackknife estimator of entropy was first described by [9]. From (2), it may be verified that, when K<∞, the bias of ${\hat{H}}_{J\;K}$ is

(7) $$E\left({\hat{H}}_{J\;K}\right)-H=O\left(n^{-2}\right).$$

Both the Miller–Madow and the jackknife estimators are adjusted versions of the plug-in. When the effective cardinality is finite, i.e., K<∞, the asymptotic normalities of both can be easily verified. A question of theoretical interest is whether these normalities still hold when the effective cardinality is countably infinite. In this paper, we give sufficient conditions for $\sqrt{n}\left({\hat{H}}_{M\;M}-H\right)$ and $\sqrt{n}\left({\hat{H}}_{J\;K}-H\right)$ to have asymptotic normalities on countably infinite alphabets and provide several illustrative examples. The rest of paper is organized as follows. Our main results for both the Miller–Madow and the jackknife estimators are given in Section 2. A small simulation study is given in Section 3. This is followed by a brief discussion in Section 4. Proofs are postponed to Section 5.

## 2. Main Results

We begin by recalling a sufficient condition due to [8] for the asymptotic normality of the plug-in estimator.

**Condition** **1.** *The distribution, $p=\{p_{k};k\geq 1\}$, satisfies*

(8) $$\begin{array}{c} \sum_{k\geq 1}p_{k}\ln^{2}p_{k}<\infty , \end{array}$$

*and there exists an integer-valued function K(n) such that, as n→∞,*

*1.*  *K(n)→∞,*
*2.*  *$K\left(n\right)/\sqrt{n}\to 0$, and*
*3.*  *$\sqrt{n}\sum_{k\geq K(n)}p_{k}\ln p_{k}\to 0$.*

Note that, by Jensen’s inequality (see e.g., [2]), (8) implies that

$$H^{2}={\left(-\sum_{k\geq 1}p_{k}\ln p_{k}\right)}^{2}\leq \sum_{k\geq 1}p_{k}\ln^{2}p_{k}<\infty ,$$

where equality holds, i.e., $H^{2}=\sum_{k\geq 1}p_{k}\ln^{2}p_{k}$, if and only if p is a uniform distribution. Thus, when (8) holds, we have H<∞. The following result is given in [8].

**Lemma** **1.** *Let $p=\{p_{k};k\geq 1\}$ be a distribution, which is not uniform, and set*

(9) $$\sigma^{2}=\sum_{k\geq 1}p_{k}\ln^{2}p_{k}-H^{2}\;and\;{\hat{\sigma }}^{2}=\sum_{k\geq 1}{\hat{p}}_{k}\ln^{2}{\hat{p}}_{k}-{\hat{H}}^{2}.$$

*If p satisfies Condition 1, then $\hat{\sigma }\overset{p}{\to }\sigma$,*

$$\frac{\sqrt{n}\left(\hat{H}-H\right)}{\sigma }\overset{L}{⟶}N\left(0,1\right),$$

*and*

$$\frac{\sqrt{n}\left(\hat{H}-H\right)}{\hat{\sigma }}\overset{L}{⟶}N\left(0,1\right).$$

The following is useful for checking when Condition 1 holds.

**Lemma** **2.** *Let $p=\{p_{k};k\geq 1\}$ and $p^{\prime }=\left\{p_{k}^{\prime };k\geq 1\right\}$ be two distributions and assume that $p^{\prime }$ satisfies Condition 1. If there exists a C>0 such that, for large enough k,*

$$p_{k}\leq Cp_{k}^{\prime },$$

*then p satisfies Condition 1 as well.*

In [8], it is shown that Condition 1 holds for $p=\{p_{k};k\geq 1\}$ with

$$p_{k}=\frac{C}{k^{2}\ln^{2}k},\;\;k=1,2,\cdots ,$$

where C>0 is a normalizing constant. It follows from Lemma 2 that any distribution with tails lighter than this satisfies Condition 1 as well.

We are interested in finding conditions under which the result of Lemma 1 can be extended to bias adjusted modifications of $\hat{H}$. Let ${\hat{H}}_{*}$ be any bias-adjusted estimator of the form

(10) $${\hat{H}}_{*}=\hat{H}+{\hat{B}}_{*},$$

where ${\hat{B}}_{*}$ is an estimate of the bias. Combining Lemma 1 with Slutsky’s theorem immediately gives the following.

**Theorem** **1.** Let $p=\{p_{k};k\geq 1\}$ be a distribution, which is not uniform, and let $\sigma^{2}$ and ${\hat{\sigma }}^{2}$ be as in (9). If Condition 1 holds and $\sqrt{n}{\hat{B}}_{*}\overset{p}{⟶}0$, then $\hat{\sigma }\overset{p}{\to }\sigma$,

$$\frac{\sqrt{n}\left({\hat{H}}_{*}-H\right)}{\sigma }\overset{L}{⟶}N\left(0,1\right),$$

*and*

$$\frac{\sqrt{n}\left({\hat{H}}_{*}-H\right)}{\hat{\sigma }}\overset{L}{⟶}N\left(0,1\right).$$

For the Miller–Madow estimator and the jackknife estimator, respectively, the bias correction term, ${\hat{B}}_{*}$, in (10) takes the form

$$\begin{array}{ccc} Miller-Madow: & \;{\hat{B}}_{M\;M} & =\frac{\hat{m}-1}{2n},\\ Jackknife: & \;{\hat{B}}_{J\;K} & =\frac{n-1}{n}\sum_{i=1}^{n}\left(\hat{H}-{\hat{H}}^{\left(i\right)}\right). \end{array}$$

Below, we give sufficient conditions for when $\sqrt{n}{\hat{B}}_{M\;M}\overset{p}{⟶}0$ and when $\sqrt{n}{\hat{B}}_{J\;K}\overset{p}{⟶}0$.

### 2.1. Results for the Miller–Madow Estimator

**Condition** **2.** *The distribution, $p=\{p_{k};k\geq 1\}$, satisfies that, for sufficiently large k,*

(11) $$\begin{array}{c} p_{k}\leq \frac{1}{a\left(k\right)b\left(k\right)k^{3}}, \end{array}$$

*where a(k)>0 and b(k)>0 are two sequences such that*

*1.*  *a(k)→∞ as k→∞, and, furthermore,*
  *(a)*  *the function a(k) is eventually nondecreasing, and*
  *(b)*  *there exists an ε>0 such that*
    (12) $$\underset{k\to \infty }{lim sup}\frac{{\left(a\left(k\right)\right)}^{2\varepsilon }}{a\left(\frac{\sqrt{k}}{{\left(a\left(k\right)\right)}^{\varepsilon }}\right)}<\infty ;$$
*2.*  (13) $$\sum_{k\geq 1}\frac{1}{kb(k)}<\infty .$$

Since this condition only requires that $p_{k}$, for sufficiently large *k*, is upper bounded in the appropriate way, we immediately get the following.

**Lemma** **3.** *Let $p=\{p_{k};k\geq 1\}$ and $p^{\prime }=\left\{p_{k}^{\prime };k\geq 1\right\}$ be two distributions and assume that $p^{\prime }$ satisfies Condition 2. If there exists a C>0 such that, for large enough k,*

$$p_{k}\leq Cp_{k}^{\prime },$$

*then p satisfies Condition 2 as well.*

We now give our main results for the Miller–Madow Estimator.

**Theorem** **2.** *Let $p=\{p_{k};k\geq 1\}$ be a distribution, which is not uniform, and let $\sigma^{2}$ and ${\hat{\sigma }}^{2}$ be as in (9). If Condition 2 holds, then $\hat{\sigma }\overset{p}{\to }\sigma$,*

$$\frac{\sqrt{n}\left({\hat{H}}_{M\;M}-H\right)}{\sigma }\overset{L}{⟶}N\left(0,1\right)$$

*and*

$$\frac{\sqrt{n}\left({\hat{H}}_{M\;M}-H\right)}{\hat{\sigma }}\overset{L}{⟶}N\left(0,1\right).$$

In the proof of the theorem, we will show that Condition 2 implies that Condition 1 holds. Condition 2 requires $p_{k}$ to decay slightly faster than $k^{-3}$ by two factors 1/a(k) and 1/b(k), where a(k) and b(k) satisfy (12) and (13) respectively. While (13) is clear in its implication on b(k), (12) is much less so on a(k). To have a better understanding of (12), we give an important situation where (12) holds. Consider the case a(n)=lnn. In this case, for any ε∈(0,0.5)

$$\frac{{\left(a\left(n\right)\right)}^{2\varepsilon }}{a\left(\frac{\sqrt{n}}{{\left(a\left(n\right)\right)}^{\varepsilon }}\right)}=\frac{{\left(\ln n\right)}^{2\varepsilon }}{0.5\ln n-\varepsilon \ln\ln n}∼\frac{{\left(\ln n\right)}^{2\varepsilon }}{0.5\ln n}⟶0.$$

We now give a more general situation, which shows just how slow a(k) can be. First, we recall the iterated logarithm function. Define $\ln^{\left(r\right)}\left(x\right)$, recursively for sufficiently large x>0, by $\ln^{\left(0\right)}\left(x\right)=x$ and $\ln^{\left(r\right)}\left(x\right)=\ln\left(\ln^{\left(r-1\right)}x\right)$ for r≥1. By induction, it can be shown that $\frac{d}{dx}\ln^{\left(r\right)}\left(x\right)={\left(\prod_{i=0}^{r-1}\ln^{\left(i\right)}\left(x\right)\right)}^{-1}$ for r≥1.

**Lemma** **4.** *The function $a\left(n\right)=\ln^{\left(r\right)}\left(n\right)$ satisfies (12) with ε=0.5 for any r≥2.*

We now give three examples.

**Example** **1.** *Let $p=\{p_{k};k\geq 1\}$ be such that for sufficiently large k,*

$$p_{k}\leq \frac{C}{k^{3}\left(\ln k\right){\left(\ln\ln k\right)}^{2+\varepsilon }},$$

*where ε>0 and C>0 are fixed constants. In this case, Condition 2 holds with a(k)=lnlnk and $b\left(k\right)=\left(\ln k\right){\left(\ln\ln k\right)}^{1+\varepsilon }/C$ in (11).*

We can consider a more general form, which allows for even heavier tails.

**Example** **2.** *Let r be an integer with r≥2 and let $p=\{p_{k};k\geq 1\}$ be such that, for sufficiently large k,*

$$p_{k}\leq \frac{C}{k^{3}\left(\prod_{i=1}^{r-1}\ln^{\left(i\right)}k\right){\left(\ln^{\left(r\right)}k\right)}^{2+\varepsilon }}$$

*where ε>0 and C>0 are fixed constants. In this case, Condition 2 holds with $a\left(k\right)=\ln^{\left(r\right)}k$ and $b\left(k\right)=\left(\prod_{i=1}^{r-1}\ln^{\left(i\right)}k\right){\left(\ln^{\left(r\right)}k\right)}^{1+\varepsilon }/C$ in (11). The fact that b(k) satisfies (13) follows by the integral test for convergence.*

It follows from Lemma 3 that any distribution with tails lighter than those in this example must satisfy Condition 2. On the other hand, the tails cannot get too much heavier.

**Example** **3.** *Let $p=\{p_{k};k\geq 1\}$ be such that $p_{k}=Ck^{-3}$, where C>0 is a normalizing constant. In this case, Condition 2 does not hold. However, Condition 1 does hold.*

### 2.2. Results for the Jackknife Estimator

For any distribution p, let $B_{n}=E\left(\hat{H}\right)-H$ be the bias of the plug-in based on a sample of size *n*.

**Condition** **3.** *The distribution, $p=\{p_{k};k\geq 1\}$, satisfies*

$$\lim_{n\to \infty }n^{3/2}\left(B_{n}-B_{n-1}\right)=0.$$

**Theorem** **3.** *Let $p=\{p_{k};k\geq 1\}$ be a distribution, which is not uniform, and let $\sigma^{2}$ and ${\hat{\sigma }}^{2}$ be as in (9). If Conditions 1 and 3 hold, then $\hat{\sigma }\overset{p}{\to }\sigma$,*

$$\frac{\sqrt{n}\left({\hat{H}}_{J\;K}-H\right)}{\sigma }\overset{L}{⟶}N\left(0,1\right)$$

*and*

$$\frac{\sqrt{n}\left({\hat{H}}_{J\;K}-H\right)}{\hat{\sigma }}\overset{L}{⟶}N\left(0,1\right).$$

It is not clear to us whether Conditions 1 and 3 are equivalent or, if not, which is more stringent. For that reason, in the statement of Theorem 3, both conditions are imposed. The proof of the theorem uses the following lemma, which gives some insight into ${\hat{B}}_{J\;K}$ and Condition 3.

**Lemma** **5.** *For any probability distribution $p=\{p_{k};k\geq 1\}$, we have*

$${\hat{B}}_{J\;K}=\frac{n-1}{n}\sum_{i=1}^{n}\left(\hat{H}-{\hat{H}}^{\left(i\right)}\right)\geq 0$$

*and*

$$E\left[{\hat{B}}_{J\;K}\right]=\left(n-1\right)\left(B_{n}-B_{n-1}\right)\geq 0.$$

We now give a condition, which implies Condition 3 and tends to be easier to check.

**Proposition** **1.** *If the distribution $p=\{p_{k};k\geq 1\}$ is such that there exists an ε∈(1/2,1) with $\sum_{k\geq 1}p_{k}^{1-\varepsilon }<\infty$, then Condition 3 is satisfied.*

We now give an example where this holds.

**Example** **4.** *Let $p=\{Ck^{-(2+\delta )};k\geq 1\},$ where δ>0 is fixed and C>0 is a normalizing constant. In this case, the assumption of Proposition 1 holds and thus Condition 3 is satisfied.*

To see that the assumption of Proposition 1 holds in this case, fix ε∈(1/2,(1+δ)/(2+δ)). Note that -(1+δ/2)<-(2+δ)(1-ε)<-1, and thus

$$\sum_{k\geq 1}p_{k}^{1-\varepsilon }=C^{1-\varepsilon }\sum_{k\geq 1}k^{-(2+\delta )(1-\varepsilon )}<\infty .$$

## 3. Simulations

The main application of the asymptotic normality results given in this paper is the construction of asymptotic confidence intervals and hypothesis tests. For instance, if p satisfies the assumptions of Theorem 2, then an asymptotic (1-α)100% confidence interval for *H* is given by

$$\begin{array}{c} \left({\hat{H}}_{M\;M}-z_{\alpha /2}\frac{\hat{\sigma }}{\sqrt{n}},{\hat{H}}_{M\;M}+z_{\alpha /2}\frac{\hat{\sigma }}{\sqrt{n}}\right), \end{array}$$

where $z_{\alpha /2}$ is a number such that $P(Z>z_{\alpha /2})=\alpha /2$ and *Z* is a standard normal random variable. Similarly, if the assumptions of Theorem 3 are satisfied, then we can replace ${\hat{H}}_{M\;M}$ with ${\hat{H}}_{J\;K}$, and if the assumptions of Lemma 1 are satisfied, then we can replace ${\hat{H}}_{M\;M}$ with $\hat{H}$. In this section, we give a small-scale simulation study to evaluate the finite sample performance of these confidence intervals.

**Figure 1:**
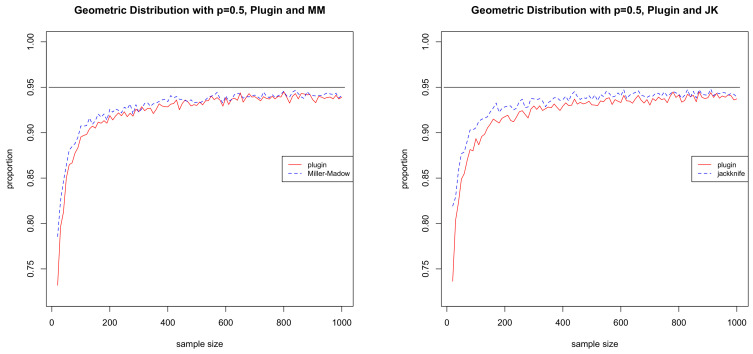

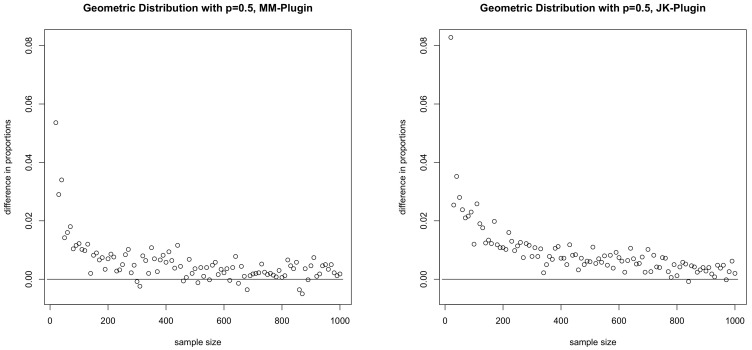
Effectiveness of the 95% confidence intervals as a function of sample size. The plot on the top left gives the proportions for the Miller–Madow and the plug-in estimators, while the one on the top right gives the proportions for the jackknife and the plug-in estimators. The horizontal line is at 0.95. The closer the proportion is to this line, the better the performance. The plot on the bottom left gives the proportion for Miller–Madow minus the proportion for the plug-in, while the one of the bottom right gives the proportion for the jackknife minus the proportion for the plug-in. The larger the value, the greater the improvement due to bias correction. Here, the horizontal line is at 0.

For concreteness, we focus on the geometric distribution, which corresponds to

$$p_{k}=p{\left(1-p\right)}^{k-1},\;k=1,2,\cdots ,$$

where p∈(0,1) is a parameter. The true entropy of this distribution is given by $H=-p^{-1}\left(p\ln p+(1-p)\ln(1-p)\right)$. In this case, Conditions 1, 2, and 3 all hold. For our simulations, we took p=0.5. The simulations were performed as follows. We began by simulating a random sample of size *n* and used it to evaluated a 95% confidence interval for the given estimator. We then checked to see if the true value of *H* was in the interval or not. This was repeated 5000 times and the proportion of times when the true value was in the interval was calculated. This proportion should be close to 0.95 when the confidence interval works well. We repeated this for sample sizes ranging from 20 to 1000 in increments of 10. The results are given in Figure 1. We can see that the Miller–Madow and jackknife estimators consistently outperform the plug-in. It may be interesting to note that, although the proofs of Theorems 1–3 are based on showing that the bias correction term approaches zero, it does not mean that the bias correction term is not useful. On the contrary, bias correction improves the finite sample performance of the asymptotic confidence intervals.

## 4. Discussion

In this paper, we gave sufficient conditions for the asymptotic normality of the Miller–Madow and the Jackknife estimators of entropy. While our focus is on the case of countably infinite alphabets, our results are formulated and proved in the case where the effective cardinality *K* may be finite or countably infinite. As such, they hold in the case of finite alphabets as well. In fact, for finite alphabets, Conditions 1–3 always hold and we have asymptotic normality so long as the underlying distribution is not uniform. The difficulty with the uniform distribution is that it is the unique distribution for which $\sigma^{2}$, as given by (9), is zero (see the discussion just below Condition 1). When the distribution is uniform, the asymptotic distribution is chi-squared with (K-1) degrees of freedom (see [6]).

In general, we do not know if our conditions are necessary. However, they cover most distributions of interest. The only distributions, which they preclude, are ones with extremely heavy tails. However, in complete generality, Conditions 1–3 may look complicated, and they are easily checked in many situations. For instance, Condition 2 always holds when, for large enough *k*, $p_{k}\leq Ck^{-3-\delta }$ for some C,δ>0, i.e., when

$$\sum_{k=1}^{\infty }k^{2}p_{k}<\infty .$$

If the alphabet X=N is the set of natural numbers, then this is equivalent to the distribution p having a finite variance. Similarly, Conditions 1 and 3 both holding is the case when, for large enough *k*, $p_{k}\leq Ck^{-2-\delta }$ for some C,δ>0, i.e., when

$$\sum_{k=1}^{\infty }kp_{k}<\infty .$$

If the alphabet X=N is the set of natural numbers, then this is equivalent to the distribution p having a finite mean.

## 5. Proofs

**Proof** **of** **Lemma** **2.** Without loss of generality, assume that C>1 and thus that lnC>0. Let f(x)=xlnx for x∈(0,1). It is readily checked that *f* is negative and decreasing for $x\in (0,e^{-1})$. Since $Cp_{k}^{\prime }\to 0$ as k→∞, it follows that $Cp_{k}^{\prime }<e^{-1}$ for large enough *k*. Now, let K(n) be the sequence that works for $p^{\prime }$ in Condition 1. For large enough *n*,

$$\begin{array}{ccc} 0 & \geq & \sqrt{n}\sum_{k\geq K(n)}p_{k}\ln p_{k}\geq C\sqrt{n}\sum_{k\geq K(n)}p_{k}^{\prime }\ln\left(Cp_{k}^{\prime }\right)\\ & \geq & C\ln\left(C\right)\sqrt{n}\sum_{k\geq K(n)}p_{k}^{\prime }+C\sqrt{n}\sum_{k\geq K(n)}p_{k}^{\prime }\ln\left(p_{k}^{\prime }\right)\\ & \geq & C\left(\ln(C)+1\right)\sqrt{n}\sum_{k\geq K(n)}p_{k}^{\prime }\ln\left(p_{k}^{\prime }\right)⟶0. \end{array}$$

Similarly, the function $g\left(x\right)=x\ln^{2}x$ for x∈(0,1) is positive and increasing for $x\in (0,e^{-2})$. Thus, there is an integer M>0 such that if k≥M, then $Cp_{k}^{\prime }<e^{-2}$ and

$$\begin{array}{ccc} 0\leq \sum_{k\geq 1}p_{k}\ln^{2}p_{k} & \leq & \sum_{k=1}^{M-1}p_{k}\ln^{2}p_{k}+C\sum_{k=M}^{\infty }p_{k}^{\prime }\ln^{2}\left(Cp_{k}^{\prime }\right)\\ & = & \sum_{k=1}^{M-1}p_{k}\ln^{2}p_{k}+C\sum_{k=M}^{\infty }p_{k}^{\prime }\ln^{2}\left(p_{k}^{\prime }\right)\\ & & \;+C\ln^{2}\left(C\right)\sum_{k=M}^{\infty }p_{k}^{\prime }+2C\ln\left(C\right)\sum_{k=M}^{\infty }p_{k}^{\prime }\ln\left(p_{k}^{\prime }\right)<\infty , \end{array}$$

as required. ☐

To prove Theorem 2, the following Lemma is needed.

**Lemma** **6.** *If Condition 2 holds, then there exists a $K_{1}>0$ such that for all $k\geq K_{1}$*

(14) $$p_{k}\leq \frac{1}{a\left(k\right)b\left(k\right)k^{3}}\leq 1-{\left(1-\frac{2}{kb(k)}\right)}^{\frac{1}{a\left(k\right)k^{2}}}.$$

**Proof.**  Observing that $e^{-x}\geq 1-x$ holds for all real *x* and that $\lim_{x\to 0}\left(1-e^{-x}\right)/x=1$, we have $e^{-2/(kb(k))}\geq 1-2/\left(kb\left(k\right)\right)$, and hence

$$\begin{array}{c} 1-{\left(1-\frac{2}{kb(k)}\right)}^{\frac{1}{a\left(k\right)k^{2}}}\geq 1-e^{-\frac{2}{a\left(k\right)b\left(k\right)k^{3}}}∼\frac{2}{a\left(k\right)b\left(k\right)k^{3}}. \end{array}$$

This implies that there is a $K_{1}>0$ such that for all $k\geq K_{1}$ (11) holds and

$$\frac{\frac{2}{a\left(k\right)b\left(k\right)k^{3}}}{1-{\left(1-\frac{2}{kb(k)}\right)}^{\frac{1}{a\left(k\right)k^{2}}}}\leq 2.$$

It follows that, for such *k*,

$$p_{k}\leq \frac{1}{a\left(k\right)b\left(k\right)k^{3}}=.5\frac{\frac{2}{a\left(k\right)b\left(k\right)k^{3}}}{1-{\left(1-\frac{2}{kb(k)}\right)}^{\frac{1}{a\left(k\right)k^{2}}}}\left[1-{\left(1-\frac{2}{kb(k)}\right)}^{\frac{1}{a\left(k\right)k^{2}}}\right]\leq 1-{\left(1-\frac{2}{kb(k)}\right)}^{\frac{1}{a\left(k\right)k^{2}}}$$

as required. ☐

**Proof** **of** **Theorem** **2.** By Theorem 1, it suffices to show that Condition 2 implies that both Condition 1 and $\sqrt{n}{\hat{B}}_{M\;M}\overset{p}{\to }0$ hold. The fact that Condition 2 implies Condition 1 follows by Example 3, Lemmas 2 and 3. We now show that $\sqrt{n}{\hat{B}}_{M\;M}\overset{p}{\to }0$.

Fix $\varepsilon_{0}\in \left(0,\varepsilon \right)$. From (12) and the facts that a(k) is positive, eventually nondecreasing, and approaches infinity, it follows that

(15) $$\begin{array}{ccc} \underset{k\to \infty }{lim sup}\frac{{\left(a\left(k\right)\right)}^{2\varepsilon_{0}}}{a\left(\frac{\sqrt{k}}{{\left(a\left(k\right)\right)}^{\varepsilon_{0}}}\right)} & = & \underset{k\to \infty }{lim sup}{\left(a\left(k\right)\right)}^{-2(\varepsilon -\varepsilon_{0})}\frac{{\left(a\left(k\right)\right)}^{2\varepsilon }}{a\left(\frac{\sqrt{k}}{{\left(a\left(k\right)\right)}^{\varepsilon_{0}}}\right)}\\ & \leq & \underset{k\to \infty }{lim sup}{\left(a\left(k\right)\right)}^{-2(\varepsilon -\varepsilon_{0})}\frac{{\left(a\left(k\right)\right)}^{2\varepsilon }}{a\left(\frac{\sqrt{k}}{{\left(a\left(k\right)\right)}^{\varepsilon }}\right)}=0. \end{array}$$

Let $K_{2}$ be a positive integer such that, for all $n\geq K_{2}$, a(n) is nondecreasing, and let $r_{n}=\left(\sqrt{n}/{\left(a\left(n\right)\right)}^{\varepsilon_{0}}\right)\vee K_{3}$, where $K_{3}=K_{1}\vee K_{2}$ and $K_{1}$ is as in Lemma 6. It follows that

$$\begin{array}{cc} E\left(\sqrt{n}{\hat{B}}_{M\;M}\right) & =\sqrt{n}E\left(\frac{\hat{m}-1}{2n}\right)\leq \frac{1}{\sqrt{n}}E\left(\hat{m}\right)=\frac{1}{\sqrt{n}}\sum_{k\geq 1}\left[1-{\left(1-p_{k}\right)}^{n}\right]\\ & \leq \frac{1}{\sqrt{n}}\sum_{k\leq r_{n}}1+\frac{1}{\sqrt{n}}\sum_{k>r_{n}}\left[1-{\left(1-\frac{2}{kb(k)}\right)}^{\frac{n}{a\left(k\right)k^{2}}}\right]=:S_{1}+S_{2}. \end{array}$$

We have

$$\begin{array}{cc} S_{1} & \leq \frac{r_{n}}{\sqrt{n}}=\left({\left(a\left(n\right)\right)}^{-\varepsilon_{0}}\right)\vee \frac{K_{3}}{\sqrt{n}}\to 0;and\\ S_{2} & \leq \frac{1}{\sqrt{n}}\sum_{k>r_{n}}\left[1-{\left(1-\frac{2}{kb(k)}\right)}^{\frac{n}{a\left(r_{n}\right)r_{n}^{2}}}\right]\leq \frac{1}{\sqrt{n}}\sum_{k>r_{n}}\left[1-{\left(1-\frac{2}{kb(k)}\right)}^{\frac{{\left(a\left(n\right)\right)}^{2\varepsilon_{0}}}{a\left(\frac{\sqrt{n}}{{\left(a\left(n\right)\right)}^{\varepsilon_{0}}}\right)}}\right]. \end{array}$$

By (15), it follows that, for large enough n,

$$S_{2}\leq \frac{1}{\sqrt{n}}\sum_{k>r_{n}}\left[1-\left(1-\frac{2}{kb(k)}\right)\right]=\frac{1}{\sqrt{n}}\sum_{k>r_{n}}\frac{2}{kb(k)}\leq \left(\sum_{k\geq 1}\frac{1}{kb(k)}\right)\frac{2}{\sqrt{n}}\to 0.$$

From here, Markov’s inequality implies that $\sqrt{n}{\hat{B}}_{M\;M}\overset{p}{⟶}0$. ☐

**Proof** **of** **Lemma** **4.** First note that $\ln^{\left(r-1\right)}\left(0.5\ln n-0.5\ln^{\left(v\right)}n\right)∼\ln^{\left(r-1\right)}\left(0.5\ln n\right)$ for any v≥2 and r≥1. This can be shown by induction on *r*. Specifically, the result is immediate for r=1. If the result is true for r=m, then for r=m+1

$$\begin{array}{ccc} \ln^{\left(m\right)}\left(0.5\ln n-0.5\ln^{\left(v\right)}n\right) & = & \ln\left(\ln^{\left(m-1\right)}\left(0.5\ln n-0.5\ln^{\left(v\right)}n\right)\right)\\ & = & \ln\left(\frac{\ln^{\left(m-1\right)}\left(0.5\ln n-0.5\ln^{\left(v\right)}n\right)}{\ln^{\left(m-1\right)}\left(0.5\ln n\right)}\right)+\ln^{\left(m\right)}\left(0.5\ln n\right)\\ & ∼ & \ln^{\left(m\right)}\left(0.5\ln n\right). \end{array}$$

It follows that for r≥2

$$\begin{array}{ccc} \lim_{n\to \infty }\frac{\ln^{\left(r\right)}n}{\ln^{\left(r\right)}\left(\sqrt{n/(\ln^{\left(r\right)}n)}\right)} & = & \lim_{n\to \infty }\frac{\ln^{\left(r\right)}n}{\ln^{\left(r-1\right)}\left(0.5\ln n-0.5\ln^{\left(r+1\right)}n\right)}\\ & = & \lim_{n\to \infty }\frac{\ln^{\left(r-1\right)}\left(\ln n\right)}{\ln^{\left(r-1\right)}\left(0.5\ln n\right)}=1, \end{array}$$

where the final equality follows by the fact that $\ln^{\left(r-1\right)}\left(x\right)$ is a slowly varying function. Recall that a positive-valued function *ℓ* is called slowly varying if for any t>0

$$\lim_{x\to \infty }\frac{\ell (xt)}{\ell (x)}=1$$

(see [11] for a standard reference). To see that $\ln^{\left(r-1\right)}\left(x\right)$ is slowly varying, note that ln is slowly varying and that compositions of slowly varying functions are slowly varying by Proposition 1.3.6 in [11]. ☐

**Proof** **of** **Lemma** **5.** Observing the convention that 0ln0=0,

$$\begin{array}{cc} \sum_{i=1}^{n}{\hat{H}}^{\left(i\right)} & =\sum_{k\geq 1}\sum_{i:X_{i}=\ell_{k}}{\hat{H}}^{\left(i\right)}\\ & =\sum_{k\geq 1}Y_{k}\left(-\frac{Y_{k}-1}{n-1}\ln\frac{Y_{k}-1}{n-1}-\sum_{j:j\geq 1,j\neq k}\frac{Y_{j}}{n-1}\ln\frac{Y_{j}}{n-1}\right)\\ & =\sum_{k\geq 1}Y_{k}\left[-\frac{Y_{k}-1}{n-1}\left(\ln\frac{Y_{k}-1}{Y_{k}}+\ln\frac{Y_{k}}{n-1}\right)-\sum_{j:j\geq 1,j\neq k}\frac{Y_{j}}{n-1}\ln\frac{Y_{j}}{n-1}\right]\\ & =\sum_{k\geq 1}Y_{k}\left[-\frac{Y_{k}-1}{n-1}\ln\frac{Y_{k}-1}{Y_{k}}+\frac{1}{n-1}\ln\frac{Y_{k}}{n-1}-\sum_{j\geq 1}\frac{Y_{j}}{n-1}\ln\frac{Y_{j}}{n-1}\right]\\ & =-\frac{1}{n-1}\sum_{k\geq 1}Y_{k}\left(Y_{k}-1\right)\ln\frac{Y_{k}-1}{Y_{k}}+\sum_{k\geq 1}\frac{Y_{k}}{n-1}\ln\frac{Y_{k}}{n-1}-\sum_{k\geq 1}Y_{k}\sum_{j\geq 1}\frac{Y_{j}}{n-1}\ln\frac{Y_{j}}{n-1}\\ & =-\frac{1}{n-1}\sum_{k\geq 1}Y_{k}\left(Y_{k}-1\right)\ln\frac{Y_{k}-1}{Y_{k}}-\left(n-1\right)\sum_{k\geq 1}\frac{Y_{k}}{n-1}\ln\frac{Y_{k}}{n-1}\\ & =-\frac{1}{n-1}\sum_{k\geq 1}Y_{k}\left(Y_{k}-1\right)\ln\frac{Y_{k}-1}{Y_{k}}-\sum_{k\geq 1}Y_{k}\left(\ln\frac{Y_{k}}{n}+\ln\frac{n}{n-1}\right)\\ & =-\frac{1}{n-1}\sum_{k\geq 1}Y_{k}\left(Y_{k}-1\right)\ln\frac{Y_{k}-1}{Y_{k}}-n\ln\frac{n}{n-1}+n\hat{H}. \end{array}$$

Therefore,

$$\begin{array}{cc} \sum_{i=1}^{n}\left(\hat{H}-{\hat{H}}^{\left(i\right)}\right)= & \frac{1}{n-1}\sum_{k\geq 1}Y_{k}\left(Y_{k}-1\right)\ln\frac{Y_{k}-1}{Y_{k}}+n\ln\frac{n}{n-1}\\ = & \frac{1}{n-1}\sum_{k\geq 1}Y_{k}\left[\left(Y_{k}-1\right)\ln\frac{Y_{k}-1}{Y_{k}}+\left(n-1\right)\ln\frac{n}{n-1}\right]\\ = & \frac{1}{n-1}\sum_{k\geq 1}Y_{k}\left[\left(n-1\right)\ln\frac{n}{n-1}-\left(Y_{k}-1\right)\ln\frac{Y_{k}}{Y_{k}-1}\right]. \end{array}$$

It suffices to show that for any y∈{1,2,⋯,n}

(16) (y-1)lny-(y-1)ln(y-1)≤(n-1)lnn-(n-1)ln(n-1).

Towards that end, first note that the inequality of (16) holds for y=1. Now, let

f(y)=(y-1)lny-(y-1)ln(y-1)

and, therefore, letting s=1-1/y,

$$f^{\prime }\left(y\right)=\ln\frac{y}{y-1}-\frac{1}{y}=\left(1-\frac{1}{y}-1\right)-\ln\left(1-\frac{1}{y}\right)=\left(s-1\right)-\ln s.$$

Since s-1≥lns for all s>0 (see e.g., 4.1.36 in [12]), f(y)≥0 for all *y*, 1<y≤n, which implies (16).

For the second part, we use the first part to get

$$0\leq E\left[\sum_{i=1}^{n}\left(\hat{H}-{\hat{H}}^{\left(i\right)}\right)\right]=nE\left[\hat{H}-H\right]-\sum_{i=1}^{n}E\left[{\hat{H}}^{\left(i\right)}-H\right]=n\left(B_{n}-B_{n-1}\right),$$

where the last equality follows from the facts that for each *i*, ${\hat{H}}^{\left(i\right)}$ is a plug-in estimator of *H* based on a sample of size (n-1) and that $E\left[{\hat{H}}^{\left(i\right)}\right]$ does not depend on *i* due to symmetry. From here, the result follows. ☐

**Proof** **of** **Theorem** **3.** By Theorem 1, it suffices to show $\sqrt{n}{\hat{B}}_{J\;K}\overset{p}{⟶}0$. Note that, by Lemma 5,

$$0\leq E\left[\sqrt{n}{\hat{B}}_{J\;K}\right]=\sqrt{n}\left(n-1\right)\left(B_{n}-B_{n-1}\right)∼n^{3/2}\left(B_{n}-B_{n-1}\right)\to 0,$$

where the convergence follows by Condition 2. From here, the result follows by Markov’s inequality. ☐

To prove Proposition 1, we need several lemmas, which may be of independent interest.

**Lemma** **7.** *Let $S_{n}$ and $S_{n-1}$ be binomial random variables with parameters (n,p) and (n-1,p), respectively. If n≥2 and p∈(0,1), then*

(17) $$E\left(S_{n}\ln S_{n}\right)=E\left(np\ln(S_{n-1}+1)\right).$$

The proof is given on page 178 in [7].

**Lemma** **8.** *Let $X_{1},\cdots ,X_{n}$ be iid Bernoulli random variables with parameter p∈(0,1). For m=1,⋯,n let $S_{m}=\sum_{i=1}^{m}X_{i}$, ${\hat{p}}_{m}=S_{m}/m$, ${\hat{h}}_{m}=-{\hat{p}}_{m}\ln{\hat{p}}_{m}$, and $\Delta_{m}=E\left[{\hat{h}}_{m}-{\hat{h}}_{m-1}\right]$. Then,*

(18) $$\Delta_{n}=E\left[{\hat{h}}_{n}-{\hat{h}}_{n-1}\right]\leq \frac{p(2-p)}{\left(n-1)[(n-2)p+2\right]}\leq \frac{2p}{\left(n-1)[(n-2)p+2\right]}.$$

**Proof.**  Applying Lemma 7 to $\Delta_{n}$ gives

$$\begin{array}{ccc} \Delta_{n} & = & p\ln\left(\frac{n}{n-1}\right)+pE\left[\ln\left(\frac{S_{n-2}+1}{S_{n-1}+1}\right)\right]\\ & = & p\ln\left(\frac{n}{n-1}\right)+pE\left[\ln\left(\frac{S_{n-2}+1}{S_{n-2}+X_{n-1}+1}\right)\right]. \end{array}$$

Conditioning on $X_{n-1}$ gives

$$\Delta_{n}=p\ln\left(\frac{n}{n-1}\right)+p^{2}E\left[\ln\left(\frac{S_{n-2}+1}{S_{n-2}+2}\right)\right].$$

Noting that f(x)=ln(x/(x+1)) is a concave function for x>0, by Jensen’s inequality,

$$\Delta_{n}\leq p\ln\left(\frac{n}{n-1}\right)+p^{2}\ln\left(\frac{(n-2)p+1}{(n-2)p+2}\right).$$

Applying the following inequalities (both of which follow from 4.1.36 in [12]) to the terms of the above expression,

$$\ln\left(\frac{x}{x-1}\right)<\frac{1}{x-1}\;\mathrm{for}\;x>1\;\mathrm{and}\;\ln\left(\frac{x}{x+1}\right)<-\frac{1}{x+1}\;\mathrm{for}\;x>0,$$

it follows that

$$\Delta_{n}\leq \frac{p}{n-1}-\frac{p^{2}}{(n-2)p+2}=\frac{p(2-p)}{\left(n-1)[(n-2)p+2\right]},$$

which completes the proof. ☐

For fixed ε>0, rewriting the upper bound of (18) gives

(19) $$\frac{2p}{\left(n-1)[(n-2)p+2\right]}=\frac{2p^{1-\varepsilon }}{n-1}\left\{\frac{p^{\varepsilon }}{(n-2)p+2}\right\}=:\frac{2p^{1-\varepsilon }}{n-1}\left\{g\left(n,p,\varepsilon \right)\right\}.$$

**Lemma** **9.** *For any ε∈(0,1) and n≥3, there exists a $p_{0}\in \left(0,1\right)$ such that g(n,p,ε) defined in (19) is maximized at $p_{0}$ and*

(20) $$0\leq g\left(n,p_{0},\varepsilon \right)=O\left(n^{-\varepsilon }\right).$$

**Proof.**  Taking the derivative of lng(n,p,ε) with respect to *p* gives

$$\frac{\partial }{\partial p}\ln g\left(n,p,\varepsilon \right)=\frac{\varepsilon }{p}-\frac{n-2}{(n-2)p+2}.$$

It is readily checked that this equals zero only at

$$p_{0}=\frac{2\varepsilon }{\left(1-\varepsilon )(n-2\right)}$$

and is positive for $0<p<p_{0}$ and negative for $p_{0}<p<1$. Thus, $p_{0}$ is the global maximum. For a fixed ε, we have

$$\begin{array}{c} g\left(n,p_{0},\varepsilon \right)=\frac{{\left(2\varepsilon \right)}^{\varepsilon }}{{\left(1-\varepsilon \right)}^{\varepsilon }{\left(n-2\right)}^{\varepsilon }\left[\frac{2\varepsilon }{\left(1-\varepsilon \right)}+2\right]}={\left(\frac{\varepsilon }{n-2}\right)}^{\varepsilon }{\left(\frac{1-\varepsilon }{2}\right)}^{1-\varepsilon }=O\left(n^{-\varepsilon }\right), \end{array}$$

as required. ☐

**Proof** **of** **Proposition** **1.** For every *k* and every m≤n, let

$$S_{m,k}=\sum_{i=1}^{m}1\left[X_{i}=\ell_{k}\right]\;\mathrm{and}\;{\hat{H}}_{m}=-\sum_{k\geq 1}\frac{S_{m,k}}{m}\ln\left(\frac{S_{m,k}}{m}\right)$$

be the observed letter counts and the plug-in estimator of entropy based on the first *m* observations. Thus, $S_{m,k}=Y_{k}$ and ${\hat{H}}_{n}=\hat{H}$. We are interested in evaluating

$$\begin{array}{cc} B_{n}-B_{n-1} & =E\left({\hat{H}}_{n}-{\hat{H}}_{n-1}\right)\\ & =\sum_{k\geq 1}E\left[\frac{S_{n-1,k}}{n-1}\ln\left(\frac{S_{n-1,k}}{n-1}\right)-\frac{S_{n,k}}{n}\ln\left(\frac{S_{n,k}}{n}\right)\right]\\ & \leq \sum_{k\geq 1}\frac{2p_{k}}{\left(n-1\right)[\left(n-2\right)p_{k}+2]}\\ & =2\sum_{k\geq 1}p_{k}^{1-\varepsilon }\frac{g(n,p_{k},\varepsilon )}{\left(n-1\right)}, \end{array}$$

where the third line follows by Lemma 8. Now, applying Lemmas 5 and 9 gives

$$\begin{array}{cc} 0\leq n^{3/2}\left(B_{n}-B_{n-1}\right) & \leq 2n^{3/2}\sum_{k\geq 1}p_{k}^{1-\varepsilon }\frac{g(n,p_{k},\varepsilon )}{\left(n-1\right)}\\ & \leq n^{3/2}\frac{g(n,p_{0},\varepsilon )}{n-1}\sum_{k\geq 1}p_{k}^{1-\varepsilon }=O\left(n^{1/2-\varepsilon }\right), \end{array}$$

which converges to zero when ε∈(1/2,1). ☐
